# Case Report: The first report of PPP2R1A mutations in mesonephric-like adenocarcinoma of endometrial carcinoma

**DOI:** 10.3389/fonc.2023.1212648

**Published:** 2023-12-04

**Authors:** Lei Cai, Chenmin Yang, Yijin Gu, Lei Dong, Weiwei Feng

**Affiliations:** ^1^ Department of Gynecology and Obstetrics, Ruijin Hospital, Shanghai Jiaotong University School of Medicine, Shanghai, China; ^2^ Department of Pathology, Ruijin Hospital, Shanghai Jiaotong University School of Medicine, Shanghai, China

**Keywords:** mesonephric-like adenocarcinoma, endometrium, endometrial carcinoma, targeted sequencing, PPP2R1A

## Abstract

We performed clinical treatment, histopathology, immunohistochemistry and molecular analyses. To compare with the published literature and have a reference overview. A 57-year-old woman and a 77-year-old woman presented with mesonephric-like adenocarcinoma of endometrium at an early clinical stage. The former had no deep myometrial infiltration and no regional lymph node involvement. The latter had deep myometrial infiltration, presence of LVSI and no regional lymph node involvement. Both of the tumor cells were positive for PAX8, GATA-3,CD-10,TTF-1,AE1/AEs,Ki67,P53 and P16 in immunohistochemical staining (IHC)Test. Primary tumors were examined for gene mutations by next generation sequencing. The former was identified KRAS mutation. The latter had KRAS,PIKCA and PPP2R1A mutations. To our knowledge, it is the first time that PPP2R1A(protein phosphatase 2,regulatory subunit A,α) mutation in MLA is reported in English literature.

## 1 Introduction

Mesonephric-like adenocarcinomas (MLAs) are described as rare tumors occurring in uterine body, especially in cervix and ovary (1–4). Unlike mesonephric carcinomas of the cervix which are derived from embryologic mesonephric (Wolffian) remnants, endometrial and ovarian MLA are thought to be derived from Müllerian substrates through a process of trans-differentiation, as they often harbor co-mutations in genes more typically affected in Müllerian tumors (5).

Endometrial carcinoma can be classified into four molecular subtypes, which have significant prognostic significance. New histological subtypes include mucinous carcinoma of the intestinal type and mesonephric-like adenocarcinoma (6). Endometrial mesonephric-like adenocarcinoma (MLA) has recently been recognized as an aggressive subtype and a rare epithelial endometrial cancer (EMC) (6). It has been added to the World Health Organization Classification of female genital tumors (7, 8). These tumors are characterized by KRAS and NRAS mutations, GATA3 and TTF1 positivity, estrogen receptor and progesterone receptor negativity (9, 10).

Mesonephric-like adenocarcinoma and endometrioid carcinoma are not distinct in general pathology examination, and differential diagnosis is usually difficult in the absence of IHC and molecular features (11). Sometimes, tumor protein 53 (TP53) mutation is associated with the possible transformation from MLA to undifferentiated carcinoma which make things more confuse (12).

Another kind of confusing is mesonephric adenocarcinoma (MA)which is distinct malignancy arising in the uterine cervix and vagina. MLA displays an aggressive biological behavior including deeper myometrial invasion, more frequent cervical stromal extension and lymph vascular or distant metastasis, more advanced stage, and higher rates of recurrence than MA (3, 13). Whether these tumors originate from the mesonephric tumors or represent Muller’s tumors very similar to the mesonephric adenocarcinoma is still controversial. They are morphologically and immunohistochemical similar to mesonephric adenocarcinomas (MA) which derived from mesonephric remnant (1). MLA association and clonality with other Muller’s tumors have been demonstrated because they have same mutations of KRAS or NRAS. It’s difficult to analysis with whole-proteomic test between MA and MLA (10).

MLA is often misdiagnosed as endometrial carcinoma.A very strange phenomenon is that when the primary lesion of the uterus is still relatively limited, the tumor appears distant metastasis (3). In comparison with more common endometrial carcinomas, endometrial MLA presents at a more advanced stage. Several case series have shown that patients with endometrial MLA have worse progression-free survival (PFS) and overall survival (OS) compared with matched cohorts of patients with serous EMC (3, 14).

Here, we report two cases of mesonephric-like adenocarcinoma(MLA) of endometrial carcinoma(EC) with excellent gene mutation and molecular analysis.

## 2 Case presentation

### 2.1 Case 1

Case 1 presented at age 57 with postmenopausal bleeding for two month and 5 years of menopause. Thickness of the endometrium was 8.8mm detected using ultrasonography. She underwent hysteroscopy. Pathology was initially reported as endometrioid type adenocarcinoma. MRI also revealed a 2.2 cm linear soft tissue lesion in the endometrial cavity. The endometrial lesion appeared not to invade the deep myometrium. PET-CT was selected without evidence peritoneal, lymph node or distant metastasis. The preoperative serum level of cancer antigen 125(CA 125) was within the normal range and cancer antigen 199 (CA 199) was a little bit higher (36.7U/ml)than normal.

Her sleep was good, bowel movements were normal and appetite was average. There was no significant weight gain or loss.

Twenty years ago, she had a right fallopian tube removal due to an ectopic pregnancy. First period was at the age of 13, a previous menstrual cycle of 7/30, average menstrual flow, and no dysmenorrhea.1-0-2-1, a healthy son born in 1986.Her family history of genetic diseases included breast cancer in her sister, lung cancer in her uncle, and lung cancer in her aunt. Her basic information is as follow: Height 159 cm, Weight: 71.3 kg,BMI: 28.32 Kg/m^2^.

Gynecological examination: Vulva:married type.Vagina:free with abnormal.Cervical:no contact bleeding.Uterine body:normal size,no tenderness.Attachment: no obvious mass, no tenderness.

Under the clinical impression of endometrial cancer, she received total hysterectomy with bilateral oophorectomy,left salpingotomy,bilateral pelvic lymph node and para abdominal aortic lymph node dissection. Pathology was finally reported as mesonephric-like adenocarcinomas measuring 1.8 cm in the greatest dimension with <50% myometrial invasion, and no substantial LVSI.Twenty-six lymph nodes were removed, no pelvic and para-aortic lymph node metastasis was found. She was diagnosed as FIGO stage IA.

After Multidisciplinary consultation and discussion with pathologist, medical oncologist, radiotherapy specialist and gynecologist, she was scheduled to receive postoperative six cycles of chemotherapy with paclitaxel and platinum-based combination therapy. Radiotherapy was performed after chemotherapy.

The patient is now 22 months postoperatively and has no signs of disease progression.

### 2.2 Case 2

Case 2 presented at age 77 with postmenopausal bleeding for two month and 30 years of menopause. Thickness of the endometrium was 4.4mm and a 35*30*34mm mass with no boundary from endometrium was detected using ultrasonography. MRI also revealed a 3.7cm mass in the posterior wall of uterus but not in endometrial cavity. PET-CT was selected and peritoneal, lymph node or distant metastasis were unfound. Level of CA125 preoperative serum was 732.4U/ml and CA199 was a little bit higher (48.5U/ml) than normal.

Her sleep was good, bowel movements were normal and appetite was average. There was no significant weight gain or loss.

A history of hyperlipidemia for more than 2 years, taking statin 20 mg qd, which is currently controlled. With a history of thyroid nodules for more than 1 year, she took Euthyrox 50 mg qd,and his condition was well controlled. After a history of cerebral infarction for more than one month, she took warfarin, and stopped paralberin 1 # qd after 2021.7.1. Denied chronic history such as diabetes and hypertension.Appendectomy and tonsillar surgery underwent 50 years before. Breast fibroma surgery underwent 40 years before.2-0-0-2, all full-term natural birth. Family history: Denial family genetic history.

Gynecological examination: Vulva: married type. Vagina: a small amount of dark red blood accumulation. Cervical: no contact bleeding.Uterine body:normal size, no tenderness.Attachment: no obvious mass, no tenderness.

Under the clinical impression of malignant tumor of uterine body, she received total hysterectomy with bilateral salpingo-oophorectomy, without bilateral pelvic lymph node and para abdominal aortic lymph node dissection, because of acute cerebral infarction two month before operation with one and 1/4 tablets of warfarin continually used.

Pathology was finally reported as a FIGO stage IB mesonephric-like adenocarcinoma measuring 3.5 cm in the greatest dimension with >50% myometrial invasion and showed some foci of LVSI.

After multidisciplinary discussion(MDT), she was recommended to receive postoperative six cycles of paclitaxel and platinum-based combination therapy followed by radiotherapy. Due to IV degree myelosuppression, TC weekly therapy(T90mg, C150mg)for 8 times were changed after TC weekly chemotherapy(T120mg, C180mg) for two times. Thereafter, radiotherapy occurred five times, protocol: PTV:50.4Gy/28Fx, PTVnd: 56 Gy/28 Fx.Due to COVID 19, brachytherapy was delayed 4 months after Extra-pelvic irradiation.

**Figure 1:**
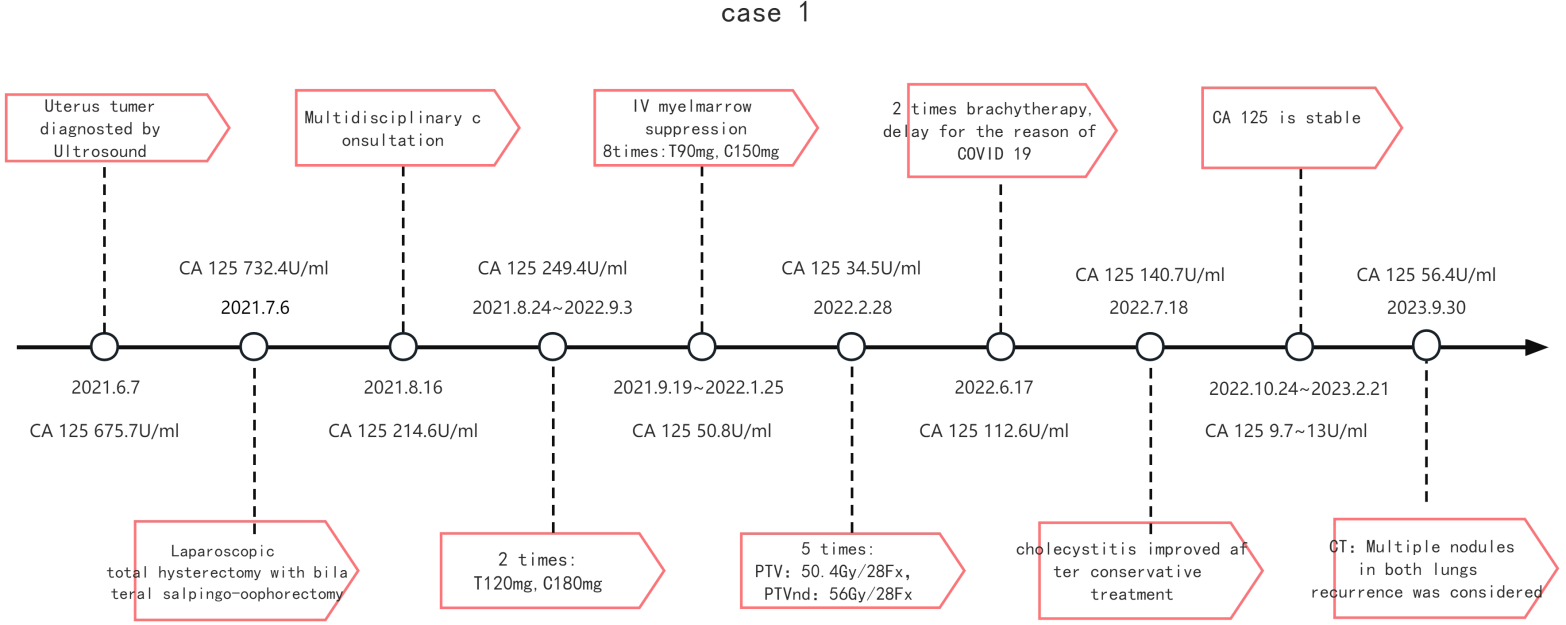
Treatment timeline of case 2.

After twenty-seven months of follow-up, the patient was found CA125 elevation (CA125 56.4U/ml).Twenty-seven months after surgery, metastasis was considered by CT examination which indicated new multiple nodules in both lungs. Further diagnosis and treatment are still in progress. (Timeline is detailed in **Figure 1**).

## 3 Pathology presentation

### 3.1 Method: NGS sequencing

Genomic DNA of FFPE sample of these two cases was extracted using the QIAamp® DNA FFPE Tissue Kit (Qiagen, Shanghai, China). DNA was then qualified with a SMA4000 spectrophotometer (Merrill Heng Tong, Beijing, China). A hybrid capture-based target NGS panel which includes 688 cancer-related genes (Beijing Genomics Institute) was applied for molecular analysis. For each case, two pools were built and sequenced on the MGISEQ-2000 sequencer using 100-bp paired-end reads. Tumor DNA samples were sequenced to an average of more than 1000× average.

### 3.2 Case 1

#### 3.2.1 Clinical pathology

Specimen type: whole uterus, right ovary, left ovary fallopian tube Tumor site: uterine. Tumor size: microscopic size 1.8cm×1.7cm×0.8cm.Histological type: adenocarcinoma, combined with immunohistochemical marker results, consistent with uterine Mesonephric-like Adenocarcinoma. Depth of infiltration: infiltration <1/2 muscle layer. Others involvement: cervical glands: (-), cervical canal interstitium (-), left side (-), right side (-), adenomsis involvement (-), uterine serous layer involvement (-),vascular invasion: no, nerve invasion: no, left peripheral margin (-), right peripheral margin (-). Left ovary: inclusion cyst, right ovary: inclusion cyst, left fallopian tube: no. Tumor involvement, right fallopian tube: none. Lymph node metastasis: 3 “left common iliac lymph nodes”, 2 “right common iliac lymph nodes”, 1 “left parailiac vascular lymph node”, 5 “right parailiac vascular lymph nodes”, 5 “left obturator lymph node”, 4 “right obturator lymph node”, 1 “abdominal aortic left lymph node”, 5 “ abdominal aorta right lymph node”, no cancer metastasis. “Presacral lymphnode” showed no carcinoma involvement.

#### 3.2.2 Histology

Microscopically, the tumor was predominated by tightly-packed, small, round tubules with single layer of cuboidal cells. Resembling the normal mesonephric tubules, densely eosinophilic secretion can be observed in some lumens. One fifth of the tumor showed papillary pattern. Nuclear pleomorphism was moderate. Mitotic figures can be easily found (5 per 10 HPF). Tumoral glands infiltrated approximately one third of thickness of the corpus.

#### 3.2.3 IHC

**Figure 2:**
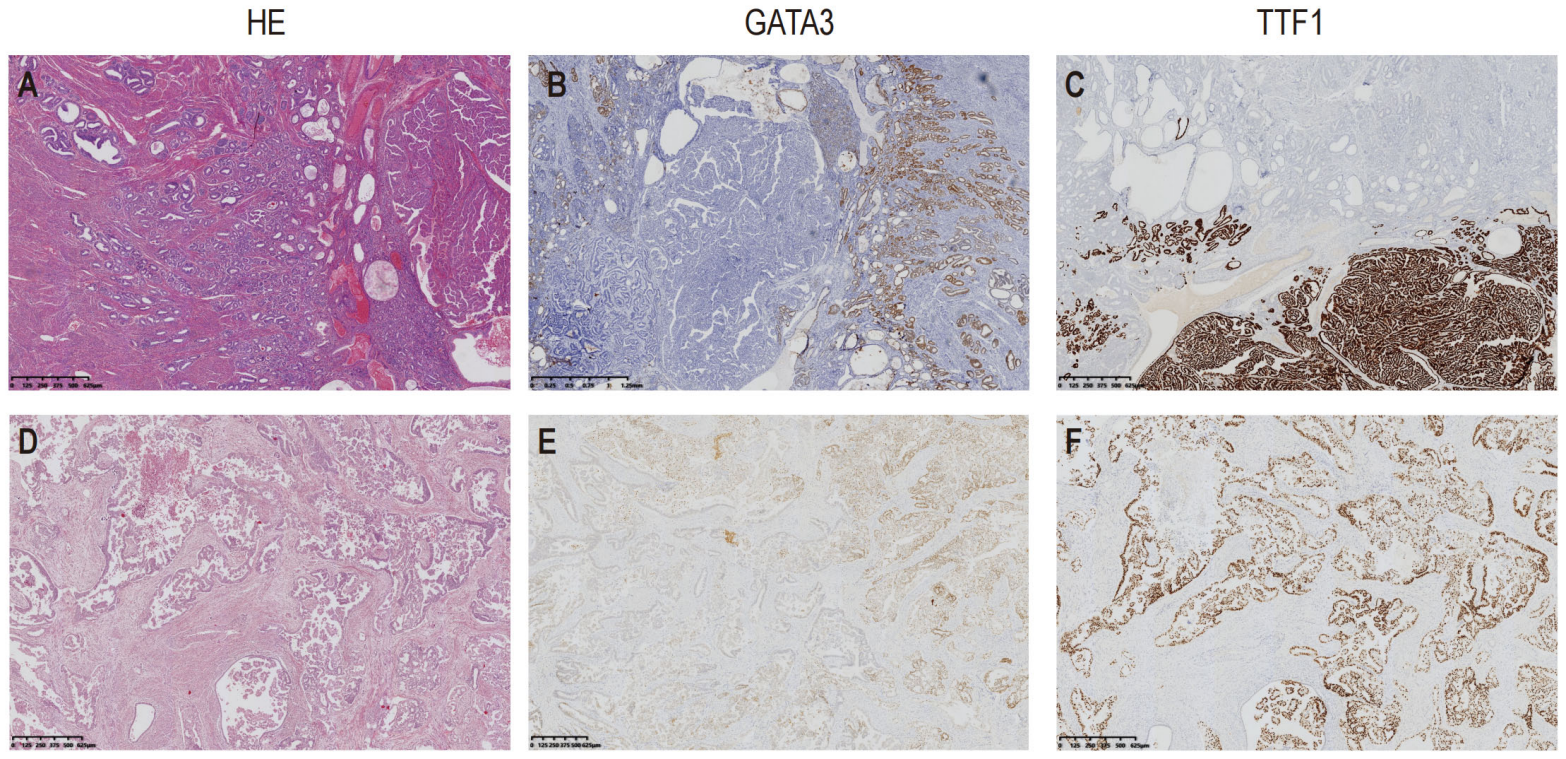
Hematoxylin and Eosin (H&E) staining, immunostaining of TTF1 and GATA3 of the two cases. Case1 showed well differentiated tubules infiltrating into the corpus. An area with papillary pattern was showed at the right side of the picture **(A)**; GATA3 mainly stained at the area of small tubules **(B)**; In contrast, TTF1 was mainly stained at the area in which the tumor presented papillary pattern **(C)**. The tumor in Case2 presented irregular ducts with papillae in the dilated lumen **(D)**; GATA3 staining was locally positive **(E)**, TTF1was showed diffusely positive stained **(F)**.

The results of immunostaining are as follow: PAX8 (+), ER (-), PR (-),P53 (wild type),WT1 (-),Vimentin (partly +),HNF1β (partly +), NapsinA (-), CD56 (partly +), AE1/AE3 (+),GATA3 (partly +), CD10 (+ luminal pattern), TTF1 (+),Calretinin (-), Ki67 (60%+), PD-L1(22C3) (CPS<1) (**Figure 2**).

#### 3.2.4 Molecular profile

**Figure 3:**
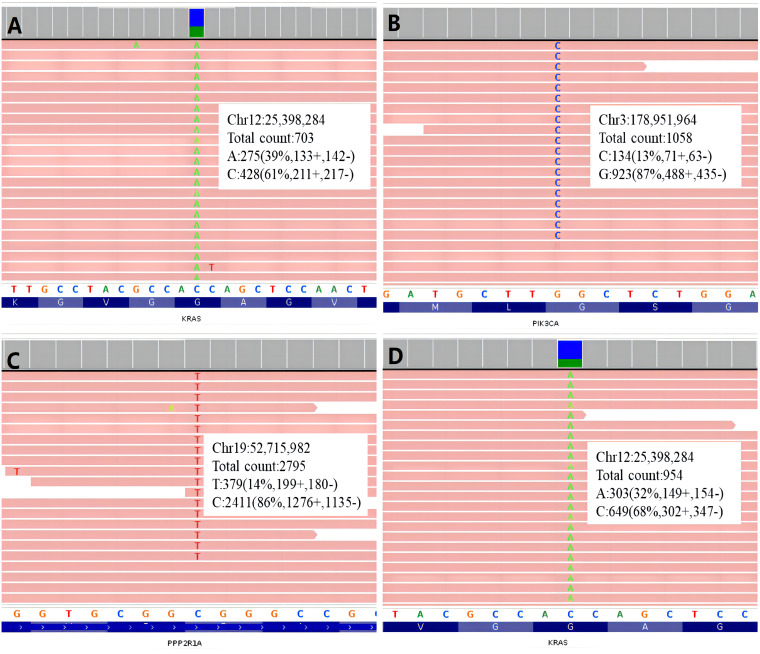
Integrative Genomics Viewer graphs of gene mutation of the two cases. **(A)** KRAS mutation of Case 1; **(B)** PIK3CA mutation of Case 2; **(C)** PPP2R1A mutation of Cases 2; **(D)** KRAS mutation of Case 2.

The only detected mutation with clinical significance was KRAS G12V (Allele Frequency: 39.30%) (**Figure 3**).

### 3.3 Case 2

#### 3.3.1 Clinical pathology

Specimen type: total uterus, bilateral ovarian fallopian tube. Tumor site: uterine. Tumor size: 3.5cm×2.5cm×2.0cm.Histological type: malignant tumor, combined with immunohistochemical marker results consistent with uterine Mesonephric-like Adenocarcinoma. Infiltration depth: infiltration> 1/2 muscle layer. Others involvement: cervical duct glands: (-), cervical canal stroma: (-), uterine serosal layer involvement: (-), vascular invasion: multiple vascular cancer plug, nerve invasion: no, cervical: mucosal chronic inflammation, left ovary: white tissue, right ovary: white tissue with inclusion cyst, left fallopian tube: mesangial paralogous cyst, right fallopian tube: no abnormalities.

#### 3.3.2 Histology

The tumor was composed of irregular ducts. In dilated ducts, glandular papillary pattern was conspicuous. Necrosis can be found in some lumen. The glands were lined by columnar cells with moderate to high grade nuclei. Half of the uterine wall was involved by tumoral ducts.

#### 3.3.3 IHC

The results of immunostaining are as follow: PAX-8(+), ER(20%+, Medium-strong staining), P16(partly +), P53(wild type), Vimentin(+), beta-Catenin(membrane+), GATA3(+), CD10(+ luminal pattern), TTF1(+), Ki67(20%+), WT-1(-), PR(-), P40(-), HNF1β(-), PD-L1(22C3) (CPS<1) (**Figure 2**).

#### 3.3.4 Molecular profile

Three missense mutations with clinical significance were detected: KRAS G12V (Allele Frequency: 30.27%), PIK3CA G1007R (Allele Frequency: 11.45%), PPP2R1A R183W (Allele Frequency: 13.11%) (**Figure 3**).

## 4 Discussion

### 4.1 Epidemiology

Mesonephric-like adenocarcinoma (MLAs) are rare tumors. The morbidity is found in 1% of endometrial cancer (13). Deolet E et al. reported 115 cases uterine-associated and 39 cases of ovarian-associated mesonephric-like carcinoma (1). The age distribution of tumors spans 26 to 91 years (1).

### 4.2 Pathogenesis

During embryonic development, females have two pairs of primordial reproductive tubes: accessory kidney (Mullerian tube) and mesonephric (Wolffian) tube. In female embryos, the Mullerian canal becomes a female reproductive canal, while the mesonephric canal is degenerated. However, the residue of the mesonephric canal may persist in the form of superepithelial inclusions along the female reproductive tract, which is known as the mesonephric remnant.

Embryo remnants are mainly found in the paraovaries and deep in the cervical stroma. Thus, Mesonephric carcinoma of the female genital tract occurs most frequently in the cervix and vagina than in the female upper genital tract.

Usually, uterine carcinoma originates from endometrium with secondary myometrial involvement and are not relative to mesonephric residual (1). Unlike mesonephric carcinomas of the cervix (embryonic mesonephric (Wolffian), the endometrial and ovarian MLA are believed to arise from Mullerian substrates through a trans-differentiation process, because they often harbor more typical genetic co-mutations in Mullerian tumors (5).

The cases of MLA associated with Mullerian lesions are all differentiated from mesonephric base (1). There are commonalities among Mullerian lesions, both with mutations of KRAS and NRAS genes (1).

### 4.3 Morphology, immunohistochemistry and molecular findings

These two cases have distinct histological morphology and molecular profile. Case 1 is a classical case with tubular pattern and KRAS mutation. KRAS mutation is characteristic in endometrial MLA. Genomic profiling in 7 endometrial or ovarian MLAs showed that all harbored canonical activating KRAS mutations (G12D or G12V) (15).

The ductal and papillary pattern of Case 2 made it necessary to be differentiated with endometrioid carcinoma, clear cell carcinoma and serous carcinoma. The immunohistochemical positivity of TTF1, GATA3, CD10 and calretinin helped to confirm the diagnosis of MLA.

Among the three gene mutations detected in Case 2, KRAS showed the highest frequency (32.27%, **Figure 2D**), two-fold more than the frequencies of the other two genes respectively (PIK3CA: 11.45%, PPP2R1A: 13.11%). It seemed, in this case, KRAS mutation was an early event, then PIK3CA and PPP2R1A mutations occurred in the subclonal tumor cells. Consequently, these two mutations showed lower frequencies. PIK3CA mutation is a relatively common finding in MLA (16).

To our knowledge, it is the first time that PPP2R1A mutation in MLA is reported in English literature. As a tumor suppressive gene, PPP2R1A inactive mutation has been recurrently found in 40% endometrial serous carcinomas. It also occurred in endometrial endometrioid carcinomas and clear cell carcinomas with much less frequency (5% and 7% respectively). PPP2R1A missense mutation R183W in Case 2 of this study is a hotspot mutation in endometrial cancer (16).

### 4.4 PPP2R1A and endometrial cancer

Protein phosphatase 2A (PP2A) is a serine-threonine phosphatase that controls a variety of cellular functions. The PPP2R1A gene is present on chromosome 19 (19q13.41) with located outside hot-spot region of residues of R182 and R183.The observation that all PPP2R1A mutations involve the α-helix repeats between the interface of subunits A and B strongly suggests that binding of both subunits is critical in cancer development (17).

PPP2R1A encodes a constant regulatory subunit of the protein phosphatase 2A holoenzyme, which is one of four major serine threonine phosphatases. It plays a role in DNA replication as a proofreader, preventing gene mutations. Studies have shown that mutations in the PPP2R1A gene are closely related to the development and progression of endometrial cancer. When PPR21A mutates, its function is impaired, leading to disruption of the MMR pathway and subsequent microsatellite instability, promoting tumor development.

The Cancer Genome Atlas (TCGA) Research Network classified all endometrial cancer types into four categories.The “copy number high” group shows increasing cell cycle deregulation (such as,CCNE1, MYC,PPP2R1A, PIKCA,ERBB2and CDKN2A) and TP53 mutations (90%) (18).

### 4.5 PPP2R1A and endometrioid-type endometrial cancer arising in adenomyosis

Hiroshi Yoshida etc. found three cases of EC-AIA had a combination of at least two molecular alterations. KRAS,TP53,PIK3CA, and PPP2R1A gene mutations, combination of at least two gene alterations in EC-AIA might contribute to carcinogenesis of adjacent adenomyosis (19). Reasons for the change from Endometriosis to Carcinogenesis become followed with interest in recent years. KRAS,TP53,PIK3CA, and PPP2R1A gene mutations test can be attempted.

### 4.6 Predictive and prognostic value of PPP2R1A

PPP2R1A plays a crucial role in many biological processes, including cell cycle control, cell apoptosis, and DNA damage repair. It is involved in tumor development, progression, and therapeutic resistance. Therefore, studying the predictive value of PPP2R1A in cancer patient prognosis is becoming increasingly important.

In recent years, it has been found that PPP2R1A mutations are high in various cancers, such as endometrial cancer. These mutations may lead to increased proliferation, invasion, and metastatic abilities of tumor cells, thereby affecting the prognosis of patients. Therefore, detecting PPP2R1A mutations in tumor samples is essential for assessing the risk of disease progression and prognosis in endometrial cancer patients.

PPP2R1A mutations are associated with poor prognosis in endometrioid and serous histological subtypes of EC patients. This may be due to:1. PPP2R1A mutations lead to impaired MMR gene function and increased microsatellite instability, making tumors more likely to undergo genetic mutations and thereby increase tumor malignancy.2. PPP2R1A mutations affect tumor cell proliferation and apoptosis, promoting tumor development.3. PPP2R1A mutations may be associated with tumor immune evasion, allowing tumor cells to escape immune surveillance and attack, increasing the risk of poor prognosis.

Although the origin of MLA is still not clear, there were evidences support at least some cases are not come from remnant mesonephric ducts, as mesonephric adenocarcinomas (MA) do. The different IHC expression patterns, the co-existence of MLA and low-grade serous adenocarcinoma showed that MLA and MA likely have different origins. Endometrial MLA might come from Mullerian type carcinoma with secondary mesonephric differentiation (20), or come from pluripotent stem cells in endometrium. The PPP2R1A R183W mutation has not been reported in MA. In contrast, its occurrence in other types of endometrial carcinoma offers further evidence to support MLA of case 2 has a non-mesonephric ducts origin.

### 4.7 Treatment and prognosis

Treatment of endometrial MLA varies widely. Almost all patients undergo upfront surgery, but adjuvant treatments differ and include radiation therapy, hormone therapy, or chemotherapy; the optimal adjuvant treatment regimen remains unknown (1). For patients with recurrent endometrial cancer, doctors need to review the original pathological section. Endometrial MLA should be well described at the time of patient’s initial surgery. Endometrium of tumor samples showed KRAS mutations, which are characteristic in patients with endometrial MLA.A meta-analysis shows that only five patients with earlier stage received postoperative chemotherapy with paclitaxel and platinum-based combination regimen from 1995 (1). Because of worse progression-free survival (PFS) and overall survival (OS) patient who was suffered with endometrial carcinoma of MLA should be given more active post-operational adjuvant chemotherapy, mainly carboplatin +paclitaxel,not only in higher stage cases but also in early stage cases. Given the rarity of these tumors, limited data exist regarding the treatment of endometrial MLA.

For the two patients in this paper, we combined all previous reported MLA cases and studies to develop the treatment plan. Although they are all early stage, they should receive six cycles of chemotherapy with paclitaxel and platinum combination and radiotherapy.

However, so far no optimal regimen can be selected. Efficacy of adjuvant radiation and/or chemotherapy are still unknown. No specific treatment have been confirmed for MLA (1). The five-year Disease Survival was about 72% for MLA of endometrium. Our cases (case1 and case 2) all received chemotherapy and radiotherapy. After 27 months, case 2 had evidence of lung recurrence. Case 1 showed no evidence of recurrence. They all still need long follow-up.

### 4.8 PPP2R1A of liquid biopsies in the future

Liquid biopsy is a method of exploring early cancer detection, diagnosis, post-treatment monitoring, and chemotherapy selection by detecting circulating tumor DNA and biomarkers in the blood. In the future, developing methods for detecting somatic mutations in tumor samples and liquid biopsies is of great significance for early screening and monitoring of tumors. Detection of somatic mutations in PPP2R1A can be selected for its clinical relevance as a biomarker for type II EC in the future (19).

## 5 Conclusions

Postoperative pathological examination of MLA, including morphology, immunohistochemistry, and especially molecular profile, are very important for the accurate diagnosis of this disease. The study of molecular profile still needs further exploration, and the origin and molecular basis of MLA are the direction of future research.

## Data availability statement

The datasets presented in this study can be found in online repositories. The names of the repository/repositories and accession number(s) can be found below: https://ngdc.cncb.ac.cn/gsa-human, HRA004253.

## Ethics statement

The Institutional Review Board (Ruijin Hospital, Shanghai Jiaotong University, School of Medicine, China) granted permission for this study (approval date: 19 June 2022) to be published on the condition that no patient identifiable data are included. Written informed consent for publication was obtained from the patient. Written informed consent was obtained from the participant/ patient(s) for the publication of this case report.

## Author contributions

WWF and LD contributed to conception and design of the study. YJG and LD finished immunal stain of tissue and genetic analysis. LC and CMY organized the database and wrote the first draft of the manuscript. WWF and LD revised the manuscript. All authors contributed to the article and approved the submitted version.
